# Abnormal Levels of Gadd45alpha in Developing Neocortex Impair Neurite Outgrowth

**DOI:** 10.1371/journal.pone.0044207

**Published:** 2012-09-06

**Authors:** Matthew R. Sarkisian, Dorit Siebzehnrubl

**Affiliations:** Department of Neuroscience, McKnight Brain Institute, University of Florida, Gainesville, Florida, United States of America; Nathan Kline Institute and New York University School of Medicine, United States of America

## Abstract

To better understand the short and long-term effects of stress on the developing cerebral cortex, it is necessary to understand how early stress response genes protect or permanently alter cells. One family of highly conserved, stress response genes is the growth arrest and DNA damage-45 (*Gadd45*) genes. The expression of these genes is induced by a host of genotoxic, drug, and environmental stressors. Here we examined the impact of altering the expression of Gadd45alpha (Gadd45a), a member of the Gadd45 protein family that is expressed throughout the developing cortices of mice and humans. To manipulate levels of Gadd45a protein in developing mouse cortex, we electroporated cDNA plasmids encoding either Gadd45a or Gadd45a shRNA to either overexpress or knockdown Gadd45a levels in the developing cortices of mice, respectively. The effects of these manipulations were assessed by examining the fates and morphologies of the labeled neurons. Gadd45a overexpression both *in vitro* and *in vivo* significantly impaired the morphology of neurons, decreasing neurite complexity, inducing soma hypertrophy and increasing cell death. Knockdown of Gadd45a partially inhibited neuronal migration and reduced neurite complexity, an effect that was reversed in the presence of an shRNA-resistant Gadd45a. Finally, we found that shRNA against MEKK4, a direct target of Gadd45a, also stunted neurite outgrowth. Our findings suggest that the expression of Gadd45a in normal, developing brain is tightly regulated and that treatments or environmental stimuli that alter its expression could produce significant changes in neuronal circuitry development.

## Introduction

Like all eukaryotic cells, developing neurons possess molecular pathways that either protect or eliminate them in the presence of cellular stressors. One family of highly conserved genes that contributes to these pathways is the growth arrest and DNA damage 45 (Gadd45) family which includes three members: alpha (Gadd45a), beta (Gadd45b) and gamma (Gadd45g) [1], [2]. Gadd45a shares ∼50% sequence homology with Gadd45b and Gadd45g [3]. Gadd45a is reported to be localized to both the nucleus and cytoplasm, and it is well known that activation or overexpression of Gadd45a inhibits cell growth and may induce cell death [3], [4]. The downstream pathways through which Gadd45a signals are diverse and include activation of various mitogen activated protein kinase (MAPK) signaling cascades, transcription factors, cell cycle regulators, and DNA repair processes (for review see: [3]). While Gadd45a expression has been detected in the early stages of mouse forebrain development [5], it remains unclear as to whether its expression persists or is inducible in developing and postnatal cerebral cortex.

In the adult brain, induction of Gadd45b, but not Gadd45a, can promote neurogenesis and neurite outgrowth [6]. Increased neuronal activity in the hippocampus induced by either electroconvulsive treatment or by exploration of novel environments has been shown to significantly increase Gadd45b expression in hippocampal neurons, which in turn leads to demethylation of specific promoter regions of growth factor genes including BDNF and FGF [6]. Like Gadd45b, Gadd45a has been implicated in DNA demethylation; however, the results of a recent study suggest that its effects within the nucleus may, in part, be mediated by its ability to bind to RNA [7]. Knockout of Gadd45a in mice can induce early neural tube defects in a small percentage of mice, an observation that raises the possibility that Gadd45a plays an active role in the developing CNS [8], [9]. Treatment of the mouse neuroblastoma cell line, N1E-115, with the HDAC inhibitor, valproic acid (VPA), has been shown to specifically upregulate Gadd45a protein expression and induce neurite outgrowth from these cells via activation of the MAP3K, MEKK4 [10]. Based on these observations, we set out to determine if changes in Gadd45a expression in developing cortex alter neuronal differentiation.

In this study, we provide evidence that Gadd45a is expressed in normal, developing cerebral cortex and that changes in its level of expression can impact the migration of developing neocortical neurons and their ability to extend normal dendritic arborizations.

## Results

### Gadd45a is Expressed in Developing Mouse and Human Cortex

**Figure 1:**
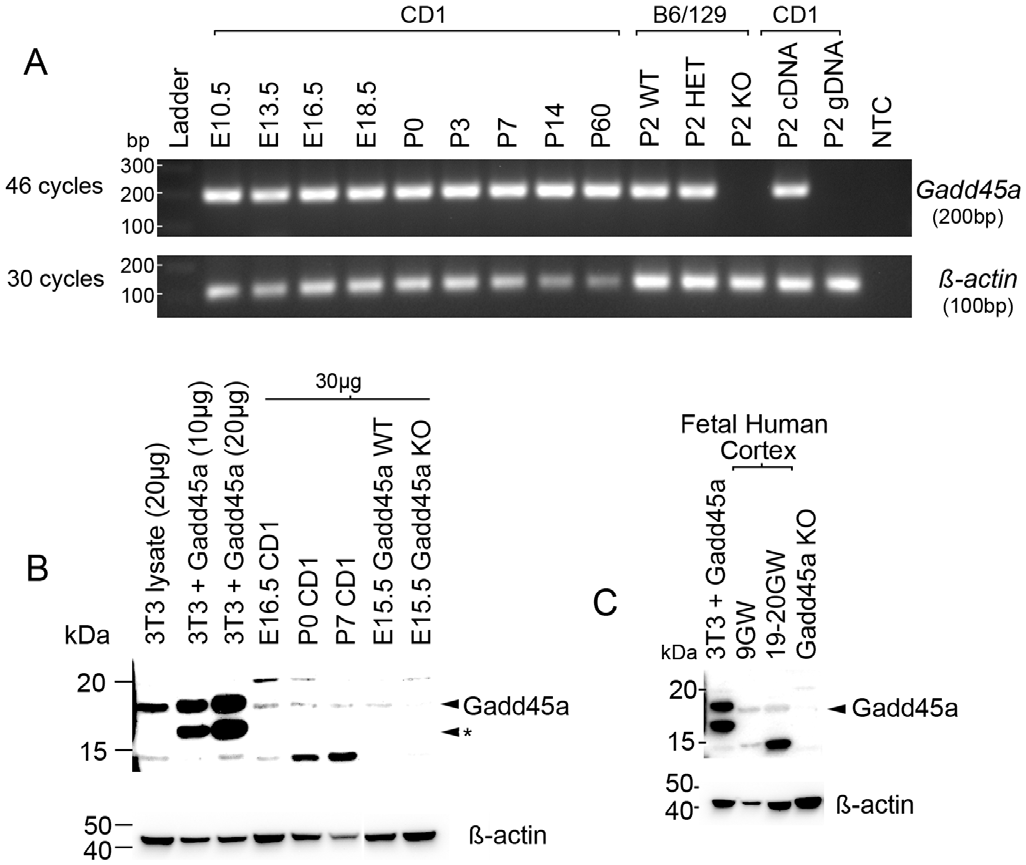
Gadd45a expression in developing cerebral cortex. (**A**) RT-PCR for Gadd45a mRNA at different ages of mouse cortex. A predicted 200 bp band for Gadd45a is detected from E10.5 to P60 in CD1 cortical extracts at 46 cycles of amplification. This band is absent in *Gadd45a^−/−^* compared to *Gadd45a^+/+^* cortex. ß-actin mRNA was used as a loading control while genomic DNA (gDNA) and no template control (NTC) were used as negative controls. (**B**) Western blot analyses of Gadd45a protein (predicted MW ∼18 kDa) in developing mouse cortex. As a positive control, we used lysates from NIH3T3 cells transfected with a plasmid expressing untagged full-length mouse Gadd45a which yielded an expected ∼18 kDa band but also a slightly lower band (arrow with asterisk) which could represent degraded or cleaved Gadd45a (see Fig. S1). Low levels of Gadd45a protein are observed at different fetal and postnatal stages of mouse CD1 cortex. There is also a band for Gadd45a WT mice that is absent in KO cortex. ß-actin is a loading control. (**C**) Western blot of different aged human forebrain lysates (9 or 19–20 gestational weeks (GW)) show a band for Gadd45a (arrowhead) at the level of our positive and negative control.

To determine if Gadd45a is expressed in developing forebrain, we analyzed forebrain tissue extracts isolated from fetal mouse and human using a combination of RT-PCR and western blot analyses (Fig. 1, Fig. S1). RT-PCR analyses of mouse cortex detected Gadd45a mRNA at all stages of cortical development through young adulthood (E10.5 to P60) (Fig. 1A). Importantly, Gadd45a mRNA was not detected in P2 and P7 *Gadd45a^−/−^* mouse cortex (Fig. 1A, Fig. S2). Western blot analyses confirmed that Gadd45a protein (predicted MW = ∼18 kDa) was detectable, albeit at low levels, in fetal and postnatal forebrain (Fig. 1B). Levels of Gadd45a protein in B6/129 lysates were difficult to detect without using long exposure times. Notably, we also detected Gadd45a at different gestational stages in developing human cortex (Fig. 1C). These data show that Gadd45a is expressed in developing human and mouse forebrain.

### Knockdown and Overexpression of Gadd45a Disrupt Neuronal Differentiation in vitro and in vivo

**Figure 2:**
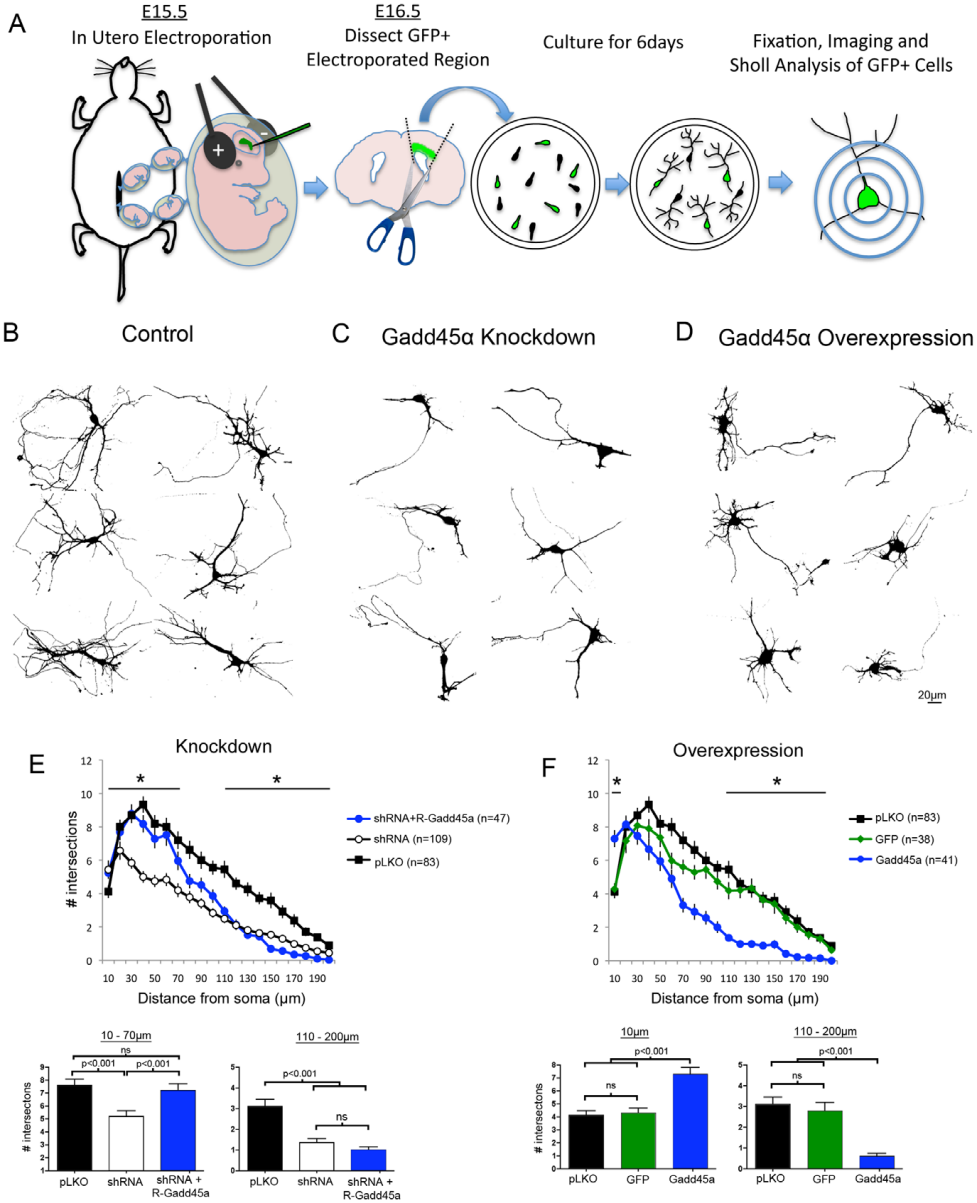
Gadd45a regulates neurite outgrowth from cortical neurons in vitro. (**A**) Method used to transfect, culture and analyze transfected cortical neurons. E15.5 mice were electroporated with cDNA encoding GFP and different Gadd45a constructs. At E16.5, GFP-positive regions of cortex were isolated, cultured 6DIV, fixed, imaged and analyzed. Sholl analyses were performed by centering concentric rings with increasing radii of 10 um over the soma center and counting the number of times the dendritic processes intersected the rings. (**B-D**) Images of GFP-positive cells converted for Sholl analyses. Examples of control (pLKO +GFP) (**B**), Gadd45a knockdown (Gadd45a shRNA + GFP) (**C**), and Gadd45a overexpressing (Gadd45a -AU1+GFP) (**D**) neurons. Scale bar = 20 µm. (**C and E**) Gadd45a knockdown results in significantly fewer neurites. (**D and F**) Gadd45a overexpression results in hypertrophied somas, and significantly fewer distal processes (∼70 µm –200 um from the soma). The significant difference within the first 10 µm is likely due to the increase in soma size (see Fig. S6). Note: Gadd45a overexpression (blue) was compared to two different controls, GFP (green) and pLKO (black, same data presented in (E)). (**E**) Electroporation of a Gadd45a-shRNA resistant construct (R- Gadd45a (blue line)) is able to significantly restore neuronal morphology compared to Gadd45a shRNA treated neurons. (**E** and **F**) Lines with asterisks indicate ranges where data was averaged for posthoc analysis (bar graphs). Sholl data was analyzed using two-way ANOVA with repeated measures while the averaged data was analyzed by Fisher’s PLSD post-hoc test. ns = not significant.

It has been reported that VPA induced upregulation of Gadd45a in neuroblastoma cells stimulates neurite outgrowth [10]. Our findings that VPA exposure increased expression of Gadd45a in a dose-dependent manner in both immortalized cells and early postnatal mouse cortex within 24 hrs of administration of the drug are consistent with this earlier report (Fig. S3). We next tested whether or not expression of Gadd45a is important for normal differentiation of neocortical neurons. Neurons were isolated from E16.5 mouse brains that had been electroporated *in utero* at E15.5 with a plasmid encoding green fluorescent protein (GFP; to visualize cell morphology) plus either a plasmid encoding Gadd45a shRNA to reduce Gadd45a levels or a plasmid encoding Gadd45a to increase Gadd45a levels. Control groups were transfected with an empty vector (pLKO) plus a vector encoding GFP or a vector encoding GFP alone. These neurons were maintained in culture for 6 days (6DIV) (Fig. 2A, Fig. S1, S4). The cultures were fixed at 6DIV and individual GFP-positive neurons and the extent of process outgrowth from the cell somas were analyzed using the Sholl method [11]. The vast majority of the GFP-positive processes analyzed were positive for the dendrite marker MAP2 (Fig. S5), but may have included axons. We found that Gadd45a shRNA significantly reduced the numbers of distal processes elaborated by these neurons (Fig. 2C and E) compared to control neurons (Fig. 2B). Similarly, increasing the levels of Gadd45a in neurons by expressing the Gadd45a plasmid reduced the numbers of processes distal to their somas, which themselves were often enlarged and irregularly shaped (Fig. 2D and F). The results of the Sholl analyses suggested that overexpression of Gadd45a increased growth of processes proximal to the neuronal somas (Fig. 2F), but it is more likely that this finding reflected an increase in the size of the somas of these cells compared to cells electroporated with either control or shRNA vectors (Fig. S6). To ensure that the shRNA effects that we observed were attributable to specific decreases in Gadd45a levels, we used site-directed mutagenesis to generate a construct encoding an shRNA-resistant Gadd45a protein (R-Gadd45a) (Fig. S4) and co-electroporated this construct and that encoding the Gadd45a shRNA. The morphologies of neurons electroporated with both R-Gadd45a and Gadd45a shRNA and grown 6DIV were similar to those of control neurons within a radius of ∼70 µm of the soma center, but these neurons did not elaborate distal processes (Fig. 2E). Our inability to rescue growth of distal processes in these cells (>70–80 µm from the soma) by co-expressing shRNA resistant Gadd45a with the Gadd45a shRNA could be due to unequal expression of the Gadd45a shRNA and R-Gadd45a constructs in the neurons, the imbalance leading to an excess Gadd45a (Fig. 2E).

**Figure 3:**
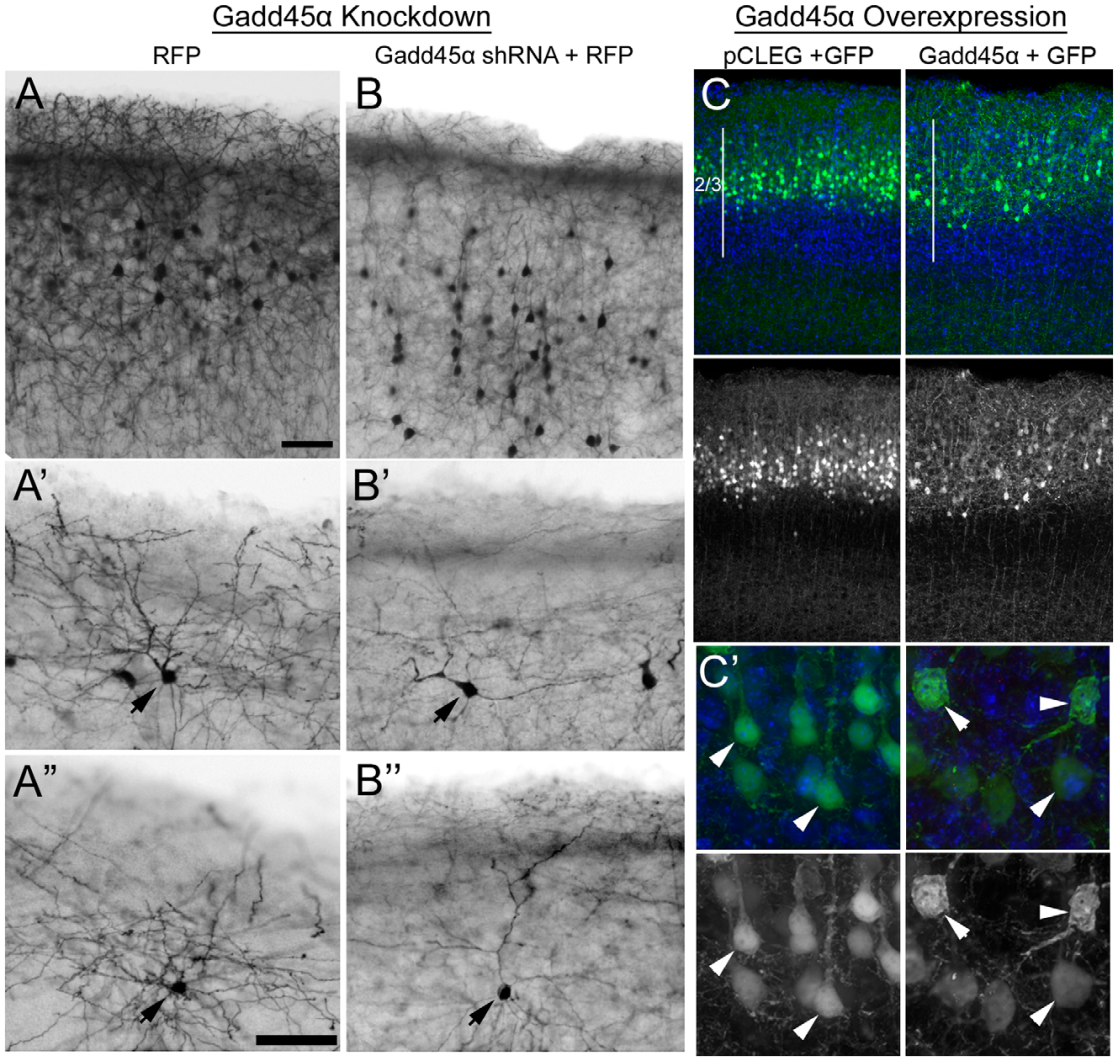
Gadd45a expression is critical for development of normal neuron morphology in neocortex. E15.5 mouse cerebral cortex was electroporated with constructs designed to reduce or increase Gadd45a levels in electroporated cells. Each construct was co-electroporated with vectors encoding red (RFP) or green (GFP) fluorescent reporter protein. RFP or GFP-positive neurons were analyzed at P14 or P10 respectively (n = 4–6 brains/group from two separate litters). (**A and B**) Although most Gadd45a shRNA-treated neurons (**B**) reached the upper layers of neocortex, there were significantly more cells distributed beneath upper layers compared to RFP alone (**A**) (also see Fig. S8). Higher magnification of control neurons (**A’ and A”**) and Gadd45a shRNA-treated (**B’ and B”**) reveals that the dendritic processes of neurons expressing Gadd45a shRNA are less arborized than those of neurons expressing RFP alone. Arrows indicate somas of RFP positive neurons. (**C**) Migration of neurons to the upper layers (2/3) of neocortex is unaffected by overexpression of Gadd45a. Left panels show neurons in control brains. Right panels show neurons overexpressing Gadd45a. (**C’**) Higher magnification images show examples of Gadd45a-AU1 neurons (right panels) with hypertrophied, multipolar shaped somas compared to control neurons (left panels). Scale bars = 50 µm (**A, A”**).

To determine if knockdown and overexpression of Gadd45a in vivo produced effects similar to those observed in vitro, we electroporated these constructs into E15.5 embryos *in utero* and examined neurons in the brains of the treated mice between P10 and P14. We found that knockdown of Gadd45a reduced the complexities of the dendritic arbors of the electroporated neurons (Fig. 3A, B) while the somas of electroporated neurons overexpressing Gadd45a were enlarged and were often multipolar in shape (Fig. 3C, Fig. S6). Thus, similar to our observations in vitro, decreased levels of Gadd45a expression in neurons in vivo reduced the extent of the dendritic arbors of these cells, while increased levels of Gadd45a expression produced neurons with irregular and hypertrophied soma morphology. In view of the observation that upregulation of Gadd45a can also trigger apoptosis [3], it is possible that the changes that we observed in the somas of neurons overexpressing Gadd45a in vitro and in vivo are early signs of impending cell death. This possibility is supported by our observation that C6-R cells overexpressing Gadd45a displayed not only stunted processes but also increased cell death (Fig. S7). In examining the cortical sections, we noted that majority of neurons either overexpressing or with reduced levels of Gadd45a reached their final migratory destination within the superficial layers of neocortex. However, analyses of these sections did reveal that a small, but significant number of neurons transfected with Gadd45a shRNA were distributed beneath the superficial layers (Fig. S8). Thus, in addition to regulating differentiation of cortical neurons, Gadd45a may have a role in promoting neuronal migration.

**Figure 4:**
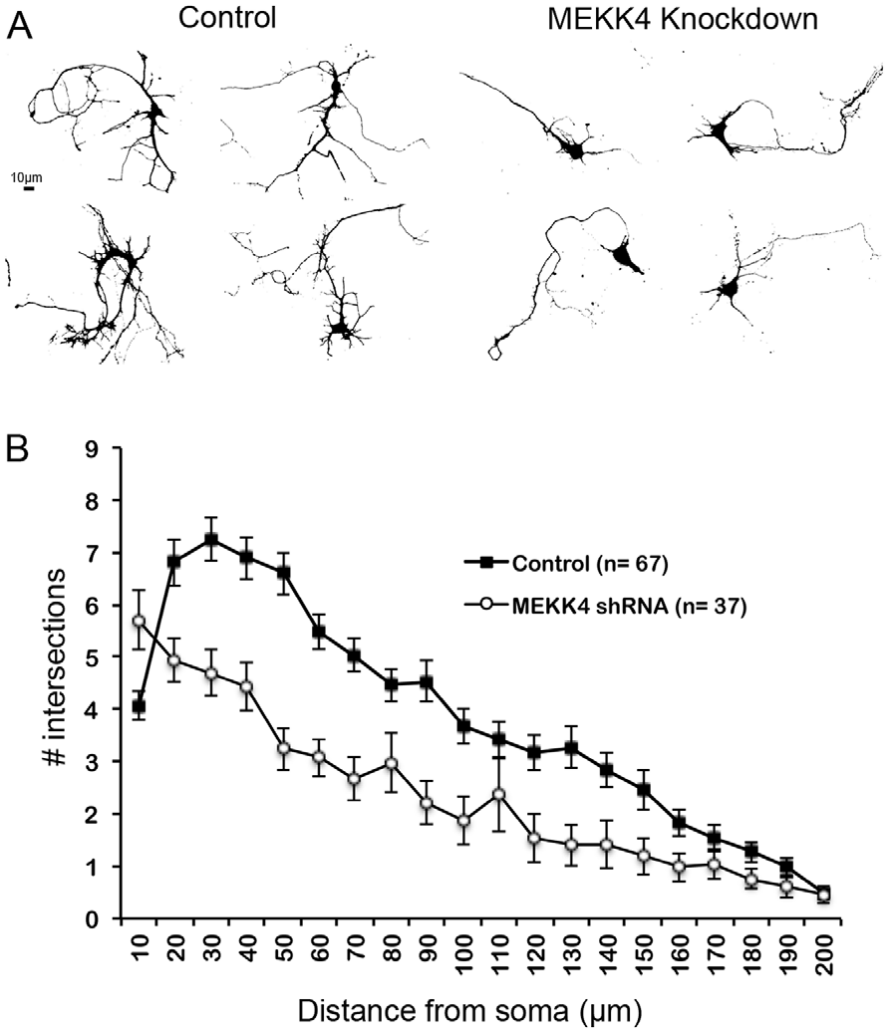
Knockdown of MEKK4 reduces neurite complexity. E15.5 mice were electroporated with cDNA encoding GFP (control) or GFP plus MEKK4 shRNA (MEKK4 knockdown). At E16.5, GFP-positive regions of cortex were isolated, cultured 6DIV, fixed, imaged and analyzed. (**A**) Example images of neurons (control and MEKK4 knockdown) converted for Sholl analyses. (**B**) Sholl analyses of the cells in (A) reveals that neurites produced by MEKK4 shRNA-transfected cells are less complex than control neurons. n = number of cells analyzed.

We next sought to identify one possible pathway through which Gadd45a could exert an influence on neuronal differentiation. Gadd45a directly binds to and activates the MAP3K, MEKK4 [2], [12], a protein reported to promote neuronal migration in developing cortex [13] and neurite outgrowth in neuroblastoma cells [10]. To determine whether knockdown of MEKK4 also disrupts neurite outgrowth of developing cortical neurons, we cultured neurons expressing control or previously characterized MEKK4 shRNA constructs [13]. Sholl analyses revealed that MEKK4 shRNA significantly reduced neurite arborizations elaborated by these neurons compared to control neurons (Fig. 4A, B). These data are consistent with the possibility that the differentiation of cortical neurons may, in part, be due to Gadd45a interactions with MEKK4.

It is noteworthy that Gadd45a levels can be detected in human blood following radiation treatment [14]. This observation in conjunction with our findings that neuron development is sensitive to changes in Gadd45a levels suggest that it may be possible to predict abnormal cortical development by monitoring Gadd45a levels in the blood. We were able to detect coordinated levels of Gadd45a mRNA in developing mouse cortex and blood (Fig. S2). Whether these levels change similarly following drug or environmental exposures requires further investigation.

Taken together, the results of our experiments suggest that the levels of Gadd45a in neurons are tightly regulated, and that the abnormal levels of expression of Gadd45a in the cortex may contribute to the arrested growth and development of cortical neurons.

## Discussion

In summary, the results of our in vitro and in vivo experiments provide strong evidence that Gadd45a plays a role in shaping the formation of the soma and dendrites of neurons in developing cortex and that either abnormal increases or decreases in the levels of Gadd45a can disrupt these developmental processes. We also found evidence that knocking down Gadd45a may induce subtle changes in neuronal migration. The mechanism(s) through which Gadd45a exerts its effects in neurons is only partially understood. In neuroblasts and other cell lines, Gadd45a has been reported to exert downstream effects on cell growth and differentiation through the MAPK pathway, including MEKK4, ERK1/2 and p38 [2], [10]. MEKK4 signaling has been shown to promote neurite outgrowth [10] and neuronal migration [13]. For example, in the N1E-115 cell line, induction of neurite outgrowth by Gadd45a was blocked in the presence of MEKK4 siRNA [10]. These observations, together with our MEKK4 data suggest that at least one Gadd45a mechanism regulating neuronal migration and arborization complexity involves MEKK4 signaling. It is likely that Gadd45a, like other Gadd45 proteins, also exerts its effects through other mechanisms of action. In adult mouse, it has been reported that electroconvulsive therapy selectively induces Gadd45b expression in brain and demethylation of the promoters of growth factor genes (e.g., BDNF and FGF) known to control hippocampal neuronal morphology [6]. Experimental evidence exists both for [15] and against [16] a role for Gadd45a in regulating gene expression by promoting DNA demethylation. The results of a recent study suggest that the effects of Gadd45a on demethylation occur via its interactions with RNA [7]. Further investigation will be required to determine the extent to which Gadd45a signaling involves downstream MAPK signaling targets and epigenetic regulation of growth factor genes. Whatever the mechanism, our data show that altered expression levels of Gadd45a can disrupt normal development of neurons.

We detected Gadd45a mRNA and protein in developing and young adult cortex. The *Gadd45a* gene normally encodes an∼18 kDa protein, however, while generating both tagged and untagged Gadd45a constructs to test our antibody specificity, we detected multiple isoforms of Gadd45a protein in cell lines expressing our constructs encoding mouse Gadd45a (Fig. S1). The presence of multiple Gadd45a isoforms is consistent with previous reports that show that Gadd45a can be cleaved and/or alternatively spliced [17], [18], [19]. For example, exposure of epithelial cells to toxins such as arsenic results in production of alternate Gadd45a isoforms [19]. It is unclear whether these Gadd45a isoforms are normally present in developing brain and if they play a role in regulating cellular activities. In addition, while all neural cell types may express low levels of Gadd45a, its presence in neurons remains to be confirmed. In our hands, antibodies that detect Gadd45a on western blots were ineffective in detecting this protein in brain sections (data not shown). The relatively low levels of Gadd45a expressed in normal brain as evidenced by western blot may have contributed to our inability to detect this protein in brain sections. Since the Gadd45 protein family is highly conserved across most cell types and has been shown to be upregulated in the brain in response to various stimuli (e.g. ultrasound [20]) it is very likely that Gadd45a is present in neurons. In addition, a recent study found that Gadd45a (endogenous or tagged) is localized to the nuclei of RKO and HEK293 cells where it colocalizes and interacts with other RNA-binding proteins [7]. Thus it would not be surprising if future studies identified similar localization in cortical neurons.

We observed that knockdown of Gadd45a in cortical neurons disrupted the outgrowth of neurites, an effect that was partially reversed by co-expression of a Gadd45a shRNA-resistant protein in these cells. These results suggest that Gadd45a may contribute to the process of neurite arborization in developing neurons. Interestingly, we found that the gross cytoarchitecture of the forebrains of Gadd45a knockout mice was comparable to that of wildtype mice (data not shown). At this point, we cannot explain this observation without further experimentation but would suggest that either the absence of Gadd45a in the knockout mice does induce differences in the morphology of neurons which are more subtle than those obtained using Gadd45a shRNA, or that the absence of Gadd45a expression in knockout mice is compensated for by other Gadd45 isoforms. This second possibility is supported by the observation that activation of *Gadd45g*, which is expressed in developing mouse and human cortex [5], [21], is sufficient to induce neuronal differentiation [22]. A third possibility is that in utero electroporation induces a more exaggerated phenotype than that obtained in knockout mice. Examples of this phenomenon have been observed for other key regulators of neuronal migration and differentiation (e.g., DCX and FMRP) [23], [24], [25].

Our findings that treatment of young mice with VPA induced rapid increases in Gadd45a protein levels in the cortex and that overexpression of Gadd45a has adverse effects on neurite arborization and survival of neurons may be clinically relevant in view of reports that children exposed to VPA in utero have a higher risk of impaired cognitive function than children exposed to other antiepileptic drugs [26]. A single, high dose of VPA (e.g. 400–800 mg/kg) is routinely administered to prenatal and early postnatal rodents by numerous research groups to induce autistic-like behaviors in these animals [27], [28], [29]. Our data suggest that these abnormal behaviors may be due, in part, to overproduction of Gadd45a in the brain. In addition to VPA, other HDAC inhibitors (e.g. trichostatin A, PCI-247810), non-steroidal anti-inflammatory drugs (e.g. indomethacin, sulindac), and various environmental stressors (e.g., hypoxia, ischemia, UV light, osmotic stress, X-rays, seizures and ultrasound) have been shown to induce Gadd45a expression [20], [30], [31], [32], [33], [34], [35]. In humans, blood levels of Gadd45a have been positively correlated with increasing levels of radiation exposure [14]. Since we were able to detect Gadd45a in both the blood and cortex of developing mice, it may be worthwhile to determine whether it is possible to detect injuries or stress-induced damage to the developing brain by monitoring changes in Gadd45a in the blood.

Collectively, our data suggest that Gadd45a signaling is required for proper neuronal morphogenesis during peak periods of neuronal maturation. It is clear that small increases or decreases in Gadd45a expression levels can alter neuron development. Because expression of Gadd45a appears to be tightly regulated in developing cortex and its expression is likely to affect brain circuitry and function, it will be important to improve our understanding of the impact that commonly used drugs and environmental stimuli that can alter Gadd45a levels have on cortical development.

## Materials and Methods

### Ethics Statement

All animal protocols were approved by and conducted in accordance with the guidelines of the University of Florida Institutional Animal Care and Use Committee. Original human fetal tissues were deposited to the Human Fetal Tissue Repository at the Albert Einstein College of Medicine (AECOM) following written maternal consent. The collection and use of these samples were approved by the Institutional Review Boards at both AECOM and Yale University. The cell lysates analyzed in this study were protein extracts of the de-identified original samples provided to the authors by P. Rakic and N. Sestan.

### Mice

Adult male and female CD1 mice were purchased from Jackson Labs and maintained in SPF conditions at the University of Florida. A line of *Gadd45a* null mice (B6/129 background) were purchased from the National Cancer Institute Mouse Depository (strain #01XAD). We generated timed-pregnant mice and considered the date of plug as embryonic day 0.5 (E0.5). Select CD1 mouse pups (n = 4–6/group) were administered intra-peritoneal (i.p.) injections of 0.9% saline or valproic acid (dosage in mg/kg where indicated; Calbiochem) dissolved in 0.9% saline at indicated ages.

### Generation of Full Length, AU1-tagged and shRNA-resistant Gadd45a cDNA Plasmids

We used a PCR-based approach to attach an AU1-tag sequence to either the C- or N-terminus (CT and NT, respectively) to full-length mouse Gadd45a. PCR products were amplified from pLE- Gadd45a -GFP (gift from Dr. Tanoue) using the primers listed (Table S1). Amplified products were digested with BglII and XhoI and cloned into pCLEG (a gift from N. Sestan [36]) that had been pre-digested with BglII and XhoI.

We also obtained pLKO.1 and pLKO.1 expressing off-target or Gadd45a-specific shRNA (Sigma). The sequence of our effective Gadd45a shRNA was CCGGCCCACATTCATCACAATGGAACTCGAGTTCCATTGTGATGAATGTGGGTTTTTG (TRC#: TRCN0000054688: Clone ID: NM_007836.1-550s1c10). We referred to this construct as shRNA#688 (characterized in Fig. S4). To construct an shRNA resistant construct (R-Gadd45a -AU1), we used the QuikChange® Lightning Site-Directed Mutagenesis Kit (Agilent Technologies) and primers listed (Table S1) to change the sense (shRNA-targeting) coding region ccacattcatcacaatggaa of the Gadd45a -AU1-NT construct into ccgcactcttctcagtggaa (mutations underlined). Characterization of both our shRNA #688 and shRNA-resistant construct is shown in the supplemental material (Fig. S4). Characterization of the MEKK4 shRNA was previously described [13].

All final constructs were sequenced to confirm their integrity.

### RNA Isolation and Reverse Transcriptase PCR (RT-PCR)

Samples of mouse cortex (n = 2–3 dissected hemispheres from 2–3 mice/timepoint) or blood were rapidly harvested, lysed and homogenized in TRIzol reagent (Invitrogen). Total RNA was isolated using RNeasy mini columns as per manufacturers instructions (Qiagen) and digested with RNase-free DNAse I (Qiagen). RNA was quantitated and cDNA was generated by reverse transcription using oligo dT primer employing the SuperScript™ First-Strand Synthesis System for RT-PCR (Invitrogen). For RT-PCR, specific primers (Table S1) were used for mouse β-actin that was amplified using Platinum Pfx Polymerase (Invitrogen) and mouse Gadd45a (same primers used in [6]) that was amplified using Advantage 2 PCR Kit (Clontech). PCR products were separated on 1.5% TAE-agarose gels and images of bands were captured and analyzed using an Alpha Innotech FluorChem Q imaging system (Cell Biosciences).

### In Utero Electroporation

To study the effects of Gadd45a knockdown or overexpression, we used in-utero electroporation (IUE) to deliver plasmid DNA into fetal cerebral cortices [13]. Typically these plasmids were co-electroporated with plasmids encoding GFP or RFP to permit visualization of cell morphology. In our hands, co-electroporation of multiple plasmids results in >90% co-transfection, similar to previous reports [23], [37], [38]. For IUE, female mice at 15.5 days into gestation were anesthesized by i.p. injections of ketamine (100 mg/kg) and xylazine (10 mg/kg) diluted in sterile 0.9% saline. The uterine horns were exposed and ∼1 µl of DNA ([0.25–2 µg/µl] mixed with 0.025% Fast-Green) was microinjected through the uterine wall into the lateral ventricles of the cerebral cortices of the mouse embryos using pulled glass capillaries. Electroporation was achieved by discharging 50V across the cortex in 5-pulse series spaced 50 msec apart using a BTX ECM 830 Square Wave Electroporator. Following injections, the dams were sutured and allowed to recover on heating pads. Electroporated embryonic mice were harvested for culture at E16.5 (see below) or transcardially perfused postnatally with saline followed by 4%PFA. Brains were removed, fixed overnight in 4%PFA, cryoprotected, frozen over liquid nitrogen and stored at -80°C until cryosectioning.

### Culture of Cell Lines and Electroporated Neurons

We used HeLa (ATCC), NIH3T3 (ATCC) and C6-R (gift from N. Sestan) [39] cell lines to examine the efficacy of Gadd45a shRNA and the expression of our Gadd45a constructs. All cells were grown in DMEM supplemented with 10% fetal bovine serum (FBS) and 1X antibiotic-anti-mycotic liquid (Invitrogen) on glass coverslips at 37°C in 5% CO_2_ and 5% relative humidity.

For culture of electroporated neurons, IUE was performed in E15.5 mice and the following day, GFP-positive regions (n = 3–5 fetal cortices/group from at least two separate experiments) were dissected from dorsal telencephalon and placed into ice-cold HBSS containing 25 mM HEPES Buffer and 0.5% glucose. Tissues were transferred into pre-warmed Trypsin LE™ solution (InVitrogen) supplemented with 10 mM HEPES and dissociated by trituration with a fire-polished glass pipette. Cells were re-suspended in Neurobasal medium (Gibco) supplemented with (to 450 ml Neurobasal we added 5 mL of 1 mM sodium pyruvate, 5 mL of 2 mM L-glutamine and 5 mL of 100X antibiotic-anti-mycotic liquid (InVitrogen), 25 mL FBS and 10 mL of B27. Cells were plated at a density of 1.5×10^5^ cells/well in 24-well plates that contained sterile glass coverslips coated with poly-ornithine (0.001%) and laminin (5 µg/ml). To promote differentiation of neurons, we replaced 50% of the culture media with media lacking serum 24 hr after seeding and repeated this replacement every other day. After 6 days in vitro (6DIV) at 37°C in 5% CO_2_, cells were fixed for 15 min in 4% PFA, rinsed, and stored in PBS at 4°C.

### Sholl Analysis of Cultured Neurons and Analysis of Soma Size

We collected photomicrographs of cultured GFP+neurons from 4–8 coverslips (typically ∼10–15 GFP+neurons/coverslip derived from at least 3–4 pooled electroporated hemispheres/group) using an Olympus IX81 spinning disc confocal microscope fitted with a 40x dry objective. All raw images of GFP+cells were re-sized to smaller higher resolution greyscale images (i.e., 18.667′′×14.222′′ at 72pix/in to 3′′×2.286′′ at 300 pix/in). Images were thresholded to high-contrast black-and-white images, and saved as tiff files using Adobe Photoshop (Version 11.01). Images were then opened in Image J64 (http://rsbweb.nih.gov/ij/) and analyzed using the Sholl Analysis plugin. We graphed the mean number of intersections of each cell’s processes with concentric rings placed every 10 µm up to 200 µm from a point marked in the soma center. Treatment groups were compared using a two-way ANOVA with repeated measures with p value <0.05 considered significant (post-hoc analyses were performed using Fisher’s PSLD test). For analysis of soma size in vitro, we measured the surface area of thresholded images of GFP+cell bodies using ImageJ64. We performed a similar analysis of soma size in vivo by tracing the cell bodies of GFP+cells from maximum projections of confocal z-stacks. Statistical analyses of soma size were compared using a Student’s t-test with p value <0.05 considered significant.

### Western Blot Analyses

Protein lysates from fetal or postnatal forebrain (n = 2–3 dissected hemispheres from 2–3 mice/timepoint) and C6-R, NIH3T3 or HeLa cells were solubilized in ice-cold 1X lysis buffer (Cell Signaling Technology) supplemented with protease and phosphatase inhibitor cocktails 1 and 2 (1∶100; Sigma) and 1 mM PMSF. Equal volumes of lysate were run on Bis-Tris gels (NuPAGE, InVitrogen) and transferred onto PVDF using an iBlot™ Dry Blotting System (InVitrogen). Blots were blocked for 1 hr in 5% NFDM in 1X Tris-buffered saline containing 0.1%Tween-20 (TBST) and incubated o/n at 4°C in primary antibodies (see below). Appropriate HRP-conjugated secondary antibodies (1∶10,000; BioRad) were detected by chemiluminescence (Pierce). Blots were performed in triplicate and images of blots were captured using an Alpha Innotech FluorChem Q imager (ProteinSimple).

### Antibodies and Histological Procedures

We used the following primary antibodies for western blot (WB), immunocytochemistry (ICC), and immunohistochemistry (IHC): mouse anti-ß-actin (1∶10,000 in WB; Sigma), chicken anti-GFP (1∶5000 in WB and IHC; Abcam), rabbit anti-Gadd45a (1∶1000 in WB, 1∶500 in ICC; Millipore), rabbit anti-Gadd45a (1∶1000 in WB; (H-165) Santa Cruz), rabbit anti-MAP2 (1∶2,000 in ICC; Millipore), goat anti-AU1 (1∶1000 in WB; Bethyl), rabbit anti-dsRed (1∶2000 in IHC; Clontech), mouse anti-GAPDH (1∶10,000 in WB; Encore Biotechnology). For IHC, appropriate, species-specific, biotin- or fluorescent-conjugated secondary antibodies (1∶400; Jackson ImmunoResearch) were used to detect the primary antibodies. For immunofluorescence, sections and/or cells were coverslipped in ProLong Gold Antifade media containing 4′,6-Diamidino-2-phenylindole dihydrochloride (DAPI) (Invitrogen).

## Supporting Information

Figure S1 **Generation and characterization of Gadd45a fusion proteins and antibodies**. (**A**) Western blot of C6-R cells transfected with either EGFP or Gadd45a-EGFP. The left blot was probed with chicken anti-GFP which yielded a band for tagged Gadd45a at ∼48 kDa (upper arrowhead). The left blot, was then stripped and re-probed using rabbit anti-Gadd45a (Millipore) (right blot) which shows only detection of Gadd45a at ∼48 kDa. Because of the low levels of expression, the lower blot was a separated for longer exposure which reveals endogenous bands for Gadd45a at ∼18 kDa (arrowhead). (**B**) Antibodies used in this study and the epitope domains of Gadd45a that are reported for generation of each antibody. (**C**) The full-length mouse Gadd45a sequence was cloned into pCLEG between the BglII and XhoI restriction sites. An AU1 tag was added to either the C-terminus (CT) or the N-terminus (NT) of Gadd45a and was separated from the Gadd45a coding region by a glycine-glycine (GG) linker. (**D**) Transfection of HeLa cells with Gadd45a -AU1 constructs shown in (C) and a Gadd45b-AU1 (CT) construct. Both Gadd45a rabbit antibodies (S Cruz and Millipore) recognize AU1-tagged Gadd45a in cells transfected with the Gadd45a constructs but importantly did not recognize Gadd45b. Although Gadd45a expression is very weak in HeLa cells, underlying endogenous Gadd45a bands are detectable with both Millipore (visible in blot) and S Cruz (not visible in blot due to intensity of AU1 signal)). When probing with the AU1 antibody (upper blot), we consistently observe a double band for Gadd45a CT tag but only a single, slightly smaller and significantly weaker band for the NT tagged protein. This observation is consistent with our observations that the Gadd45a bands detected by both of the Gadd45a antibodies (S Cruz and Millipore) were slightly smaller in size (kDa) for Gadd45a (NT) compared to Gadd45a (CT). (**E**) Our interpretation of the results shown in (**D**) suggests that there is a site for post-translational cleavage or processing (scissors) of Gadd45a located near the beginning of the N-terminus.
(TIF)

Figure S2 **Gadd45a is simultaneously expressed in both cortex and blood**. RT-PCR detection of Gadd45a mRNA in samples from P7 WT, HET and KO forebrain and blood. Samples run in the lanes of cortex and blood are derived from the same animals. Note the loss of expression in KO samples. Although weakly expressed in blood, ß-actin levels is shown as a loading control. The number of amplification cycles is also shown.
(TIF)

Figure S3 **Valproic acid (VPA)-induced upregulation of Gadd45a**. (**A**) Western blot of NIH3T3 cells treated with saline or increasing concentrations (in mM) of VPA. Blots were probed with an anti-Gadd45a antibody that shows an increase in Gadd45a with increasing concentrations of VPA. (**B**) Western blot of cortical lysates (n = 4 hemispheres/lane) 24 hr after exposure to either 50 or 200 mg/kg of VPA at P1 show a dose-dependent increase in Gadd45a.
(TIF)

Figure S4 **Identification of shRNA specific for Gadd45a knockdown and development of a Gadd45a cDNA resistant to shRNA**. (**A**) HeLa cells were transfected with different control and shRNA constructs (Sigma). Compared to controls and construct #690, construct #688 dramatically reduced Gadd45a-EGFP expression. A plasmid expressing monomeric RFP (mRFP) was used as a transfection control. Bar = 20 µm. (**B**) Western blot analyses of cells transfected as in (**A**) confirmed that shRNA #688 effectively reduces levels of Gadd45a. mRFP and ß-actin were transfection and loading controls, respectively. (**C**) Design of an shRNA-resistant R-Gadd45a-AU1 (NT) construct. Within the cDNA encoding region targeted by shRNA #688, we mutated five base pairs (red) using site-directed mutagenesis without altering the endogenous amino acid sequence. (**D**) Examination of the performance of the Gadd45a resistant construct. HeLa cells were transfected with the indicated AU1-tagged Gadd45a constructs in the presence or absence of shRNA #688. Western blot results show that shRNA#688 specifically reduced levels of both AU1-tagged Gadd45a (CT and NT) proteins (black arrows) but does not reduce levels of the shRNA-resistant R-Gadd45a-AU1 (NT) (red arrows). GFP and ß-actin served as transfection and loading controls, respectively.
(TIF)

Figure S5 **Co-localization of MAP2 and GFP in cultured electroporated neurons**. Examples of control (left column) and Gadd45a shRNA(right column) transfected neurons expressing GFP. Both cells extend MAP2 (red) and GFP-positive processes (arrowheads) from the cell body. Nuclei are labeled with DAPI (blue) in the merged channel.
(TIF)

Figure S6 **Effect of Gadd45a knockdown and overexpression on neuronal soma size**. (**A**) Quantification of soma surface area from electroporated neurons grown 6DIV. Compared to pLKO+GFP (Control) and Gadd45a shRNA #688 (shRNA), Gadd45a overexpression led to increased soma size. (**B**) Increased soma surface area in Gadd45a overexpression (pCLEG-Gadd45a-AU1+GFP) compared to Control (pCLEG+GFP) in layer 2/3 neurons.
(TIF)

Figure S7 **Gadd45a overexpression disrupts morphology and survival of C6-R cells**. C6-R cells (derived from a modified rat glioma cell line) were transfected with vectors encoding EGFP or Gadd45a-EGFP. (**A**) C6-R cells normally display an elongated bipolar shape (2 examples are shown). In contrast, cells transfected with Gadd45a-EGFP induces many of the cells to become stunted with multipolar cell bodies (6 example cells are shown). Vertical scale bar = 50 µm (**B**) Quantification of the average cell lengths between EGFP or Gadd45a-EGFP transfected cells. (**C** and **D**) Compared to control cells (**C**), many cells overexpressing Gadd45a-EGFP (**D**) displayed pyknotic nuclei colocalized with EGFP (arrows). Nuclei are labeled with DAPI.
(TIF)

Figure S8 **Gadd45a knockdown disrupts migration to the superficial layers of neocortex**. E15.5 mice were electroporated with vectors encoding RFP alone (control) or RFP plus Gadd45a shRNA #688 (Gadd45a shRNA). The brains were fixed and immunostained for RFP at P14. (**A**) Examples of immunostained sections from control and Gadd45a shRNA. Compared to control, we observed more transfected RFP positive cells (arrows) distributed below the superficial layers (i.e, within the brackets) after Gadd45a shRNA. (**B**) The percent of cells distributed beneath the upper layers of cortex in control (n = 19 sections from 3 brains) and Gadd45a shRNA (n = 20 sections from 3 brains). Data were compared by a Student’s t-test.
(TIF)

Table S1 **Primers used to generate different Gadd45a constructs and for RT-PCR analysis.**
(DOCX)
