# Supplementary figures and images for: Abnormal Levels of Gadd45alpha in Developing Neocortex Impair Neurite Outgrowth
Source: PLoS One. 2012 Sep 6;7(9):e44207. doi: 10.1371/journal.pone.0044207 (PMC3435417; doi:10.1371/journal.pone.0044207)

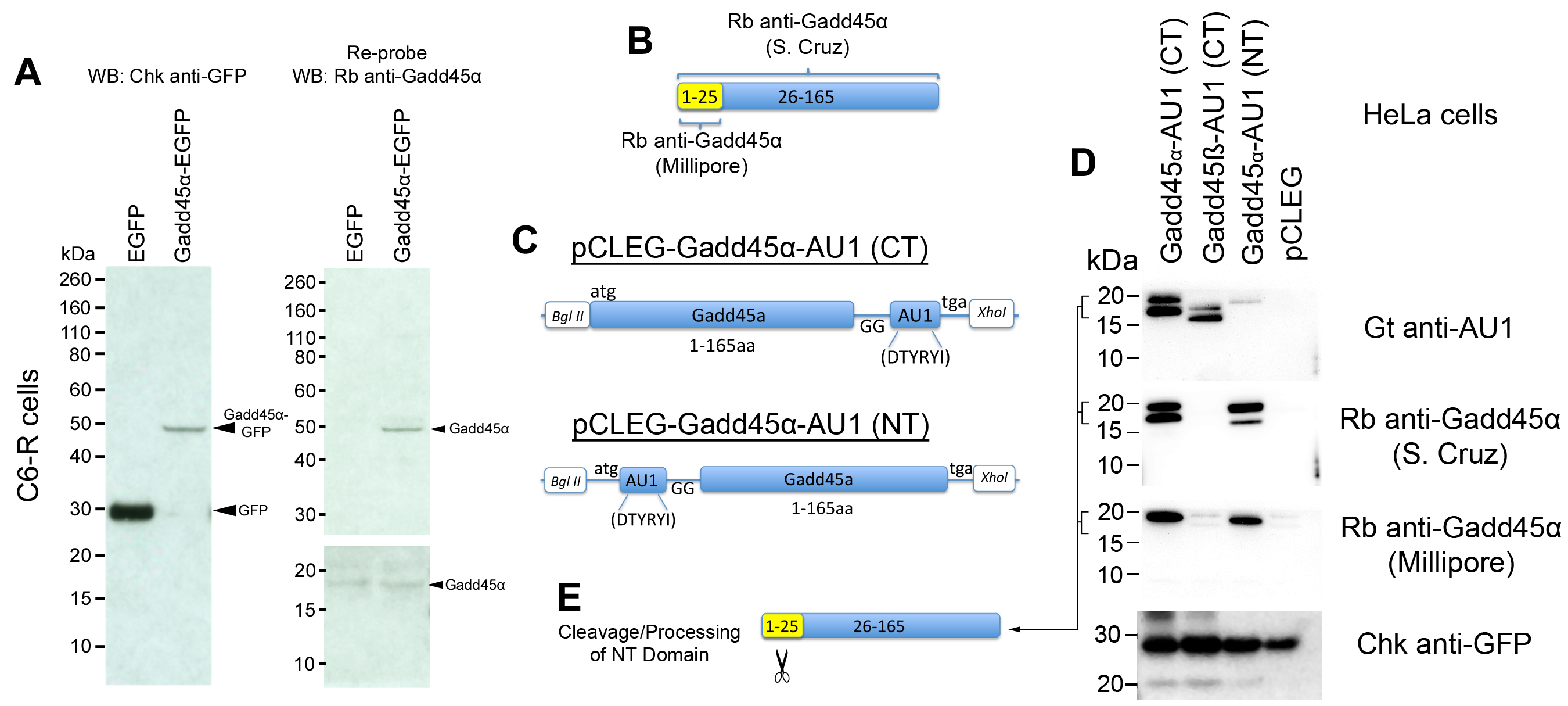

Supplement: Figure S1 — Generation and characterization of Gadd45a fusion proteins and antibodies. (A) Western blot of C6-R cells transfected with either EGFP or Gadd45a-EGFP. The left blot was probed with chicken anti-GFP which yielded a band for tagged Gadd45a at ∼48 kDa (upper arrowhead). The left blot, was then stripped and re-probed using rabbit anti-Gadd45a (Millipore) (right blot) which shows only detection of Gadd45a at ∼48 kDa. Because of the low levels of expression, the lower blot was a separated for longer exposure which reveals endogenous bands for Gadd45a at ∼18 kDa (arrowhead). (B) Antibodies used in this study and the epitope domains of Gadd45a that are reported for generation of each antibody. (C) The full-length mouse Gadd45a sequence was cloned into pCLEG between the BglII and XhoI restriction sites. An AU1 tag was added to either the C-terminus (CT) or the N-terminus (NT) of Gadd45a and was separated from the Gadd45a coding region by a glycine-glycine (GG) linker. (D) Transfection of HeLa cells with Gadd45a -AU1 constructs shown in (C) and a Gadd45b-AU1 (CT) construct. Both Gadd45a rabbit antibodies (S Cruz and Millipore) recognize AU1-tagged Gadd45a in cells transfected with the Gadd45a constructs but importantly did not recognize Gadd45b. Although Gadd45a expression is very weak in HeLa cells, underlying endogenous Gadd45a bands are detectable with both Millipore (visible in blot) and S Cruz (not visible in blot due to intensity of AU1 signal)). When probing with the AU1 antibody (upper blot), we consistently observe a double band for Gadd45a CT tag but only a single, slightly smaller and significantly weaker band for the NT tagged protein. This observation is consistent with our observations that the Gadd45a bands detected by both of the Gadd45a antibodies (S Cruz and Millipore) were slightly smaller in size (kDa) for Gadd45a (NT) compared to Gadd45a (CT). (E) Our interpretation of the results shown in (D) suggests that there is a site for post-translational cleavage [file pone.0044207.s001.tif]

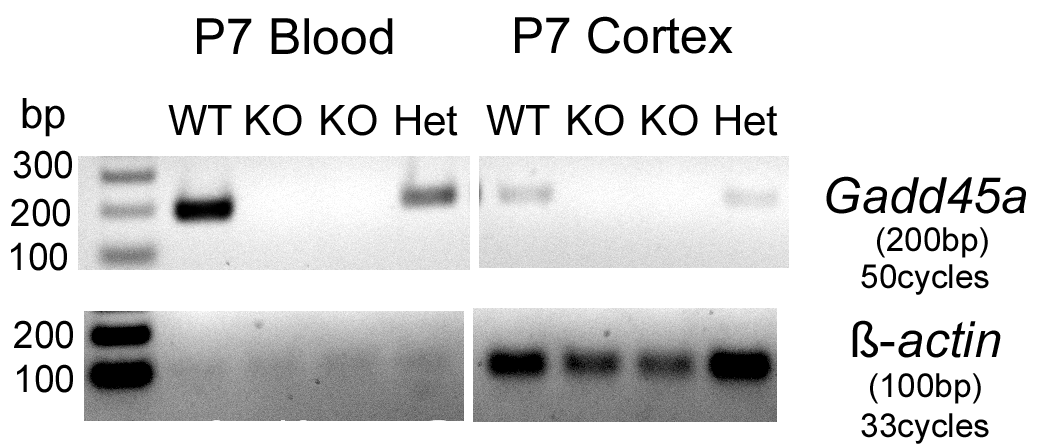

Supplement: Figure S2 — Gadd45a is simultaneously expressed in both cortex and blood. RT-PCR detection of Gadd45a mRNA in samples from P7 WT, HET and KO forebrain and blood. Samples run in the lanes of cortex and blood are derived from the same animals. Note the loss of expression in KO samples. Although weakly expressed in blood, ß-actin levels is shown as a loading control. The number of amplification cycles is also shown. (TIF) [file pone.0044207.s002.tif]

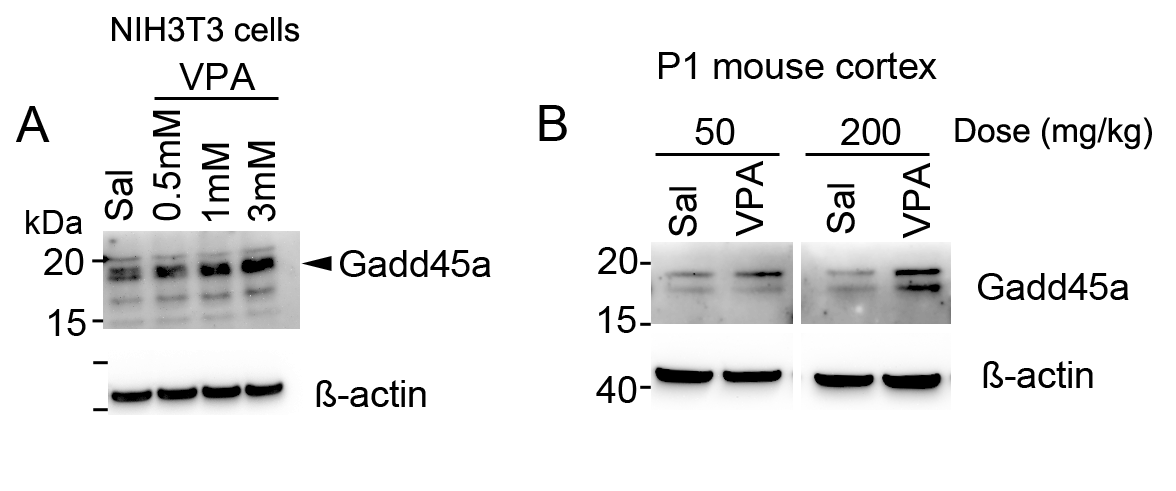

Supplement: Figure S3 — Valproic acid (VPA)-induced upregulation of Gadd45a. (A) Western blot of NIH3T3 cells treated with saline or increasing concentrations (in mM) of VPA. Blots were probed with an anti-Gadd45a antibody that shows an increase in Gadd45a with increasing concentrations of VPA. (B) Western blot of cortical lysates (n = 4 hemispheres/lane) 24 hr after exposure to either 50 or 200 mg/kg of VPA at P1 show a dose-dependent increase in Gadd45a. (TIF) [file pone.0044207.s003.tif]

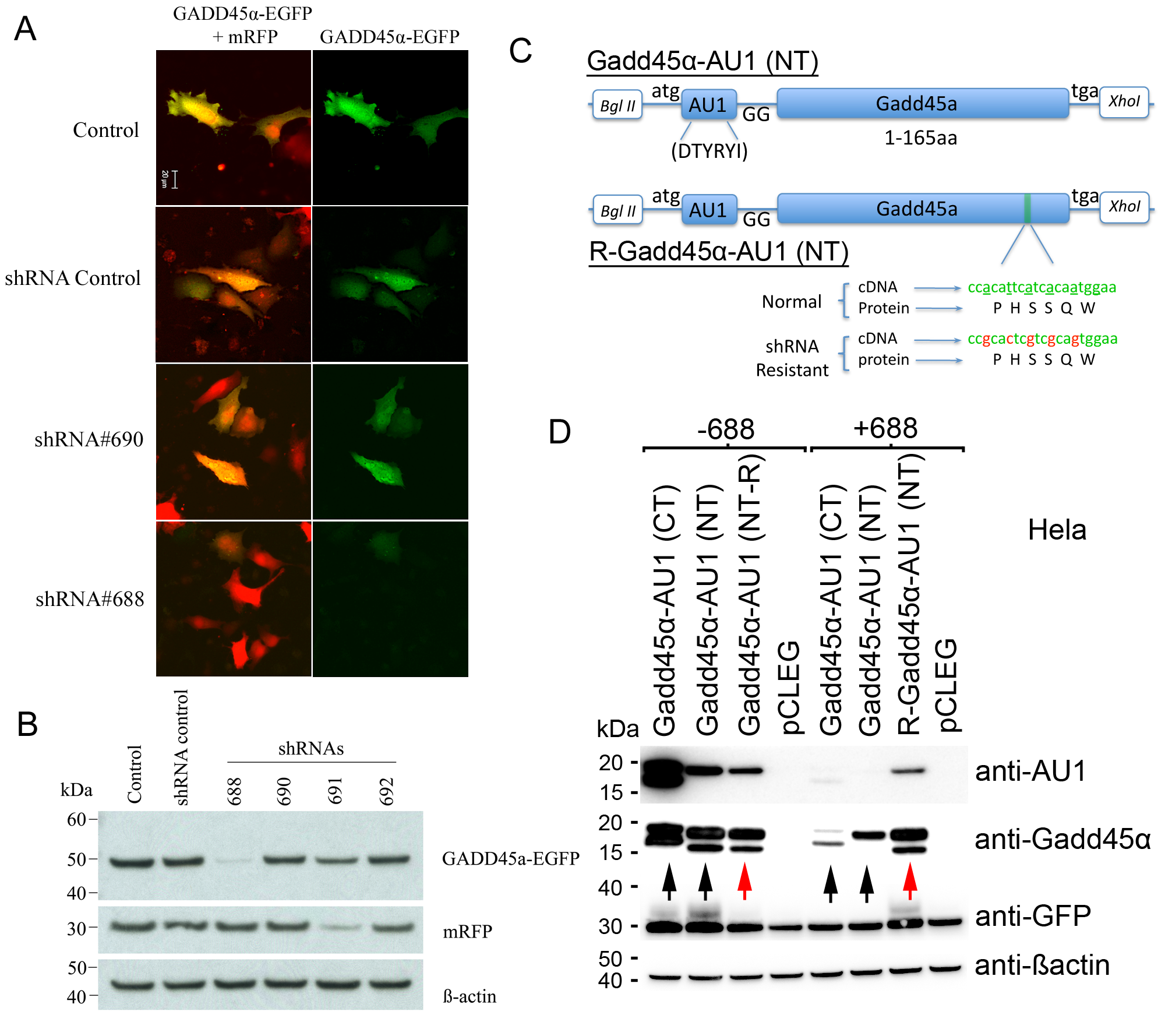

Supplement: Figure S4 — Identification of shRNA specific for Gadd45a knockdown and development of a Gadd45a cDNA resistant to shRNA. (A) HeLa cells were transfected with different control and shRNA constructs (Sigma). Compared to controls and construct #690, construct #688 dramatically reduced Gadd45a-EGFP expression. A plasmid expressing monomeric RFP (mRFP) was used as a transfection control. Bar = 20 µm. (B) Western blot analyses of cells transfected as in (A) confirmed that shRNA #688 effectively reduces levels of Gadd45a. mRFP and ß-actin were transfection and loading controls, respectively. (C) Design of an shRNA-resistant R-Gadd45a-AU1 (NT) construct. Within the cDNA encoding region targeted by shRNA #688, we mutated five base pairs (red) using site-directed mutagenesis without altering the endogenous amino acid sequence. (D) Examination of the performance of the Gadd45a resistant construct. HeLa cells were transfected with the indicated AU1-tagged Gadd45a constructs in the presence or absence of shRNA #688. Western blot results show that shRNA#688 specifically reduced levels of both AU1-tagged Gadd45a (CT and NT) proteins (black arrows) but does not reduce levels of the shRNA-resistant R-Gadd45a-AU1 (NT) (red arrows). GFP and ß-actin served as transfection and loading controls, respectively. (TIF) [file pone.0044207.s004.tif]

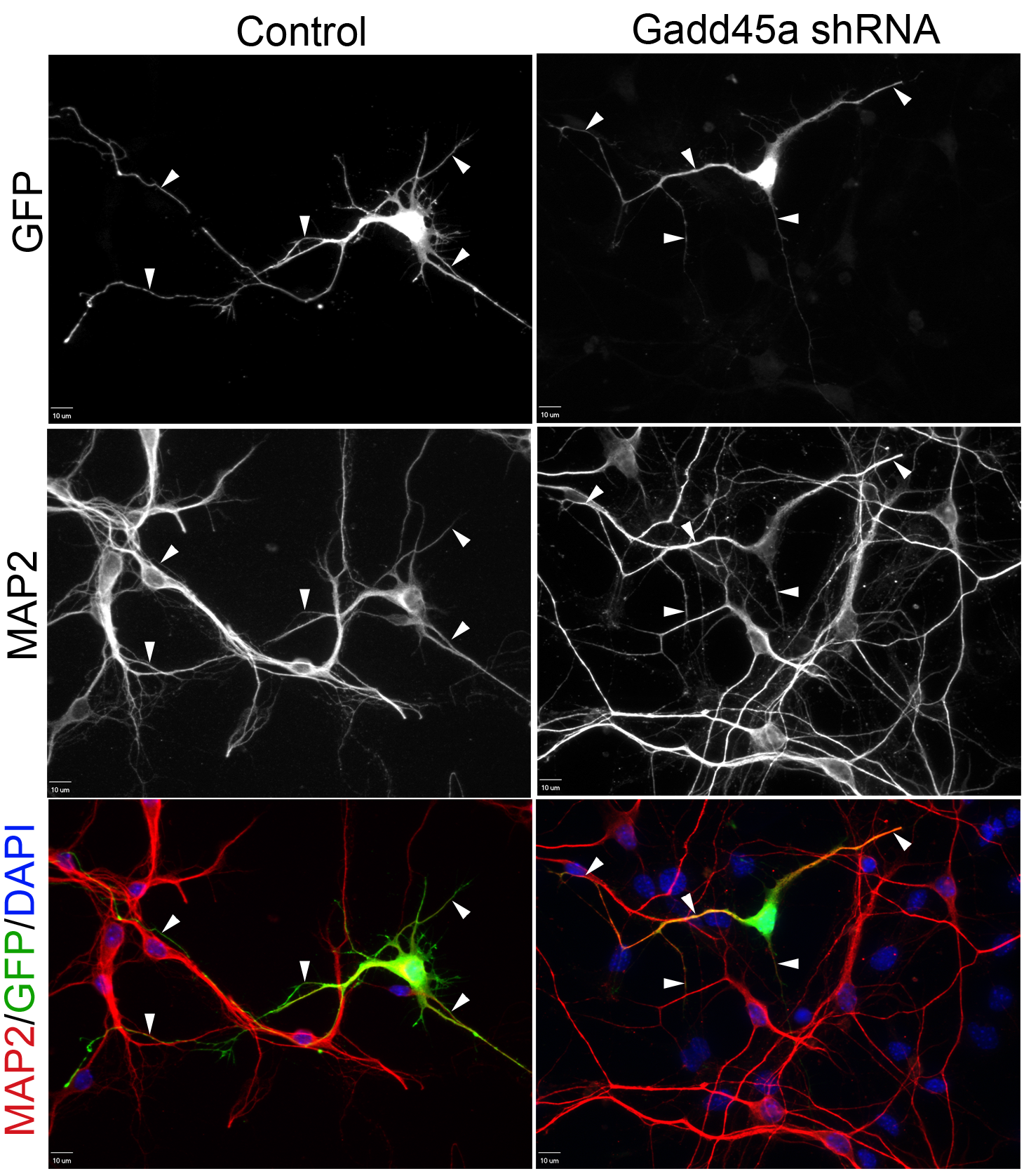

Supplement: Figure S5 — Co-localization of MAP2 and GFP in cultured electroporated neurons. Examples of control (left column) and Gadd45a shRNA(right column) transfected neurons expressing GFP. Both cells extend MAP2 (red) and GFP-positive processes (arrowheads) from the cell body. Nuclei are labeled with DAPI (blue) in the merged channel. (TIF) [file pone.0044207.s005.tif]

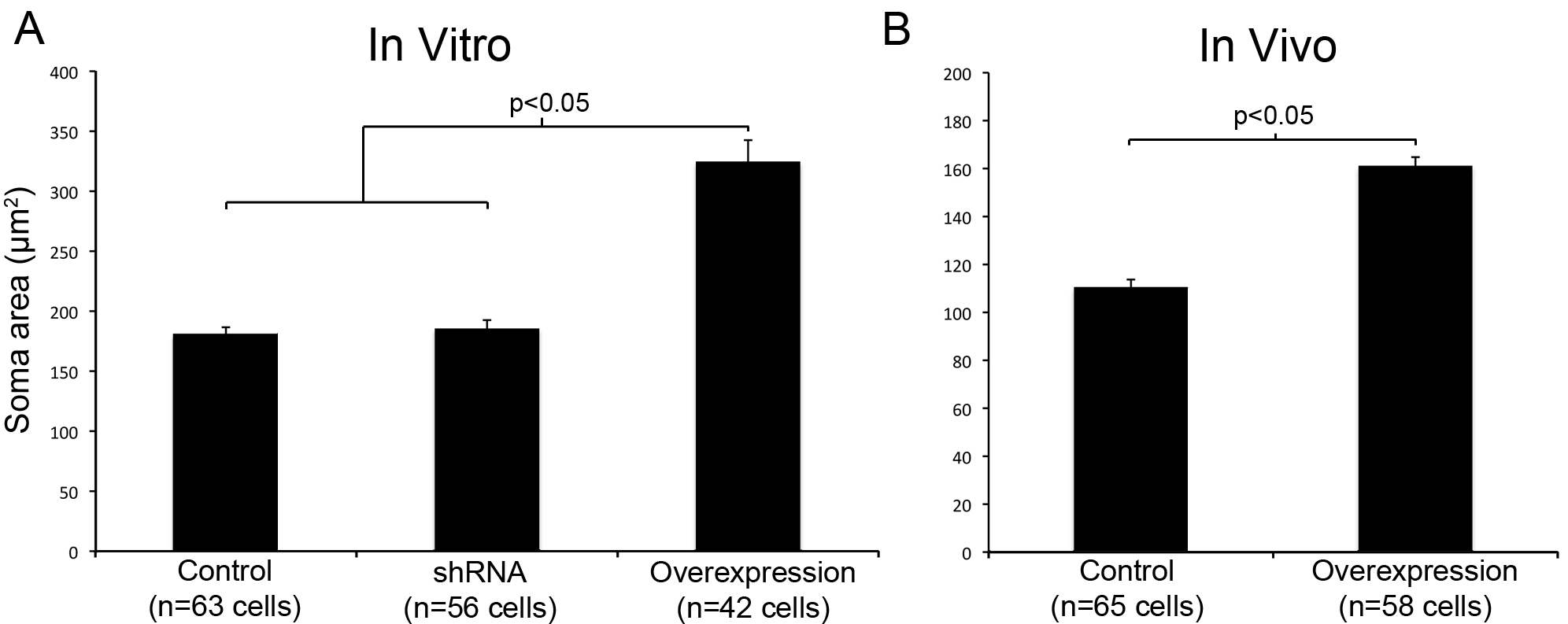

Supplement: Figure S6 — Effect of Gadd45a knockdown and overexpression on neuronal soma size. (A) Quantification of soma surface area from electroporated neurons grown 6DIV. Compared to pLKO+GFP (Control) and Gadd45a shRNA #688 (shRNA), Gadd45a overexpression led to increased soma size. (B) Increased soma surface area in Gadd45a overexpression (pCLEG-Gadd45a-AU1+GFP) compared to Control (pCLEG+GFP) in layer 2/3 neurons. (TIF) [file pone.0044207.s006.tif]

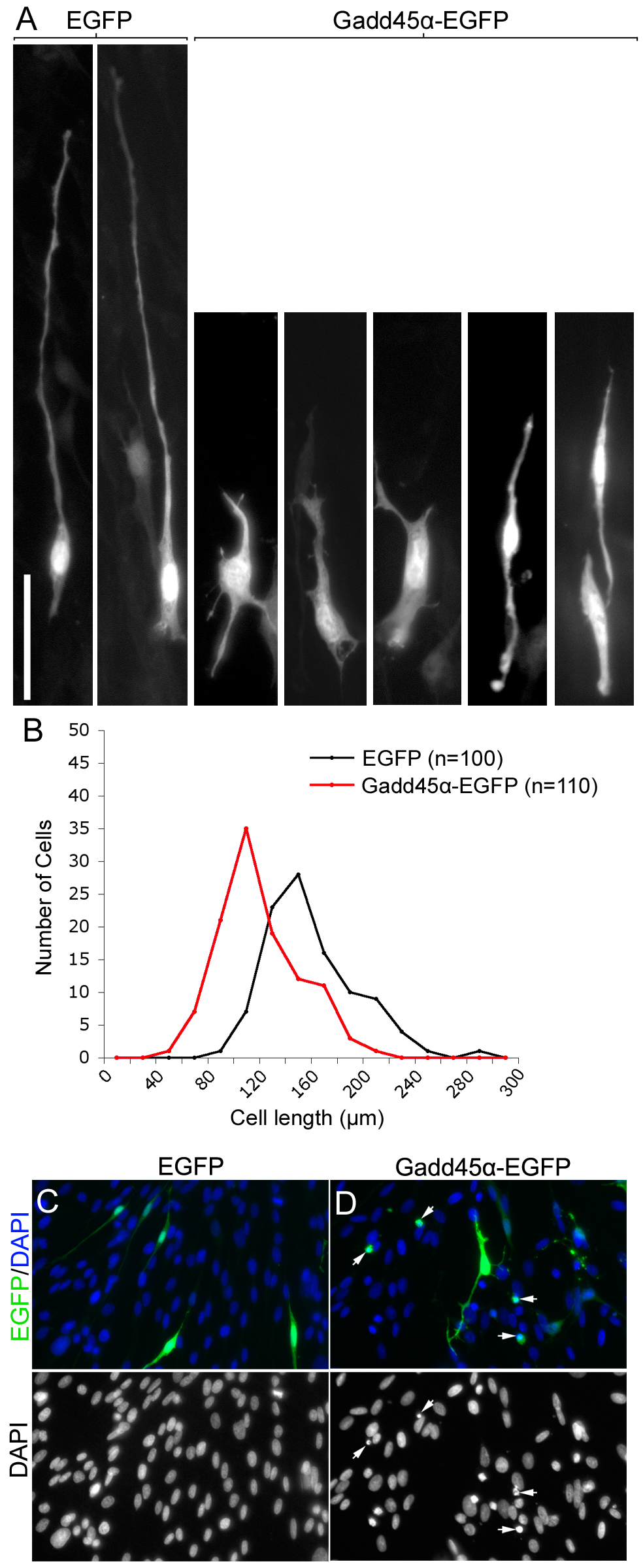

Supplement: Figure S7 — Gadd45a overexpression disrupts morphology and survival of C6-R cells. C6-R cells (derived from a modified rat glioma cell line) were transfected with vectors encoding EGFP or Gadd45a-EGFP. (A) C6-R cells normally display an elongated bipolar shape (2 examples are shown). In contrast, cells transfected with Gadd45a-EGFP induces many of the cells to become stunted with multipolar cell bodies (6 example cells are shown). Vertical scale bar = 50 µm (B) Quantification of the average cell lengths between EGFP or Gadd45a-EGFP transfected cells. (C and D) Compared to control cells (C), many cells overexpressing Gadd45a-EGFP (D) displayed pyknotic nuclei colocalized with EGFP (arrows). Nuclei are labeled with DAPI. (TIF) [file pone.0044207.s007.tif]

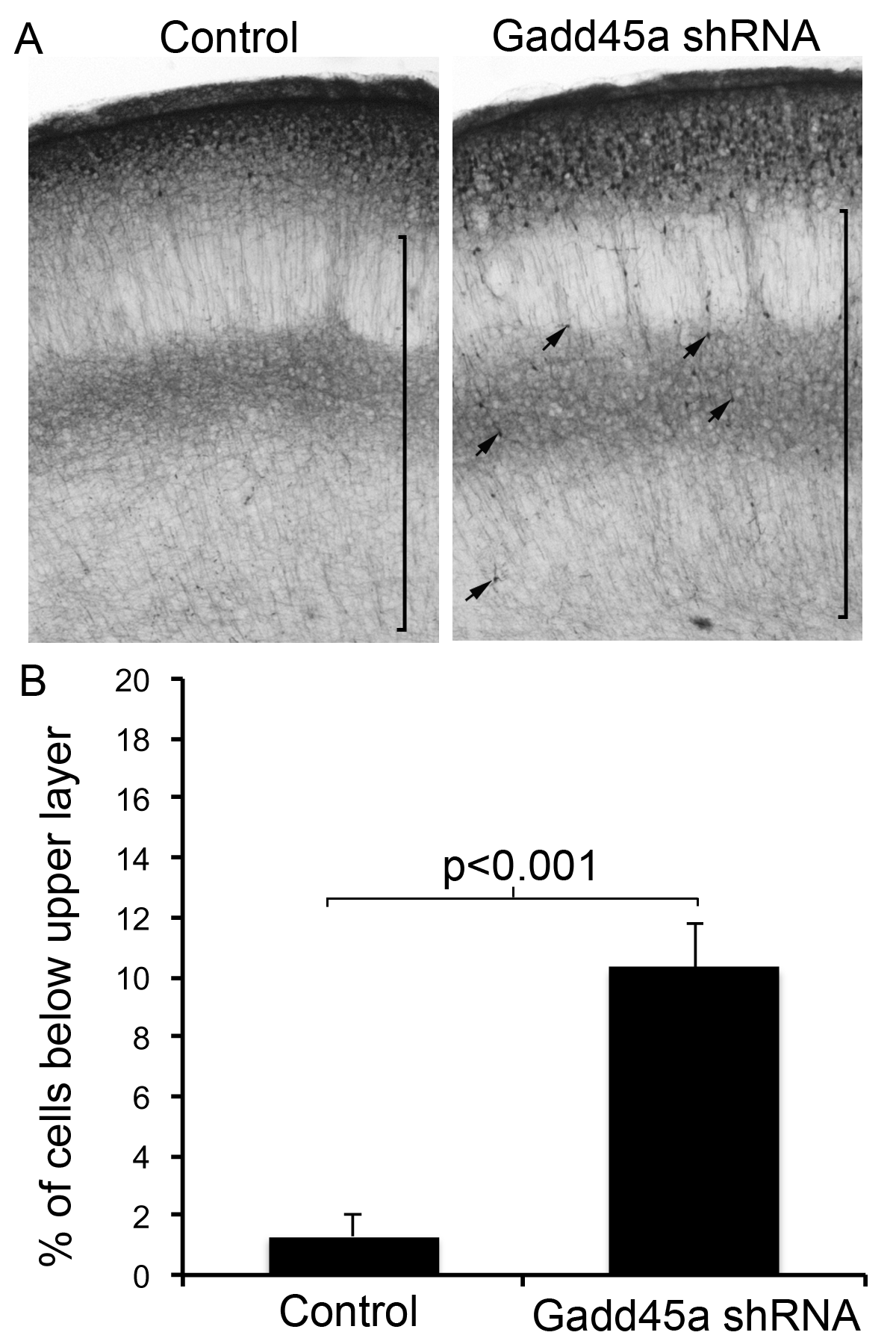

Supplement: Figure S8 — Gadd45a knockdown disrupts migration to the superficial layers of neocortex. E15.5 mice were electroporated with vectors encoding RFP alone (control) or RFP plus Gadd45a shRNA #688 (Gadd45a shRNA). The brains were fixed and immunostained for RFP at P14. (A) Examples of immunostained sections from control and Gadd45a shRNA. Compared to control, we observed more transfected RFP positive cells (arrows) distributed below the superficial layers (i.e, within the brackets) after Gadd45a shRNA. (B) The percent of cells distributed beneath the upper layers of cortex in control (n = 19 sections from 3 brains) and Gadd45a shRNA (n = 20 sections from 3 brains). Data were compared by a Student’s t-test. (TIF) [file pone.0044207.s008.tif]
