# Supplementary material for: Abnormal Levels of Gadd45alpha in Developing Neocortex Impair Neurite Outgrowth
Source: PLoS One. 2012 Sep 6;7(9):e44207. doi: 10.1371/journal.pone.0044207 (PMC3435417; doi:10.1371/journal.pone.0044207)
Supplement: Table S1 — Primers used to generate different Gadd45a constructs and for RT-PCR analysis. (DOCX) [file pone.0044207.s009.docx]

| **#** | **Construct/Template** | **Primer** | **Primer Sequence (5’ to 3’)** |
| --- | --- | --- | --- |
| 1 | Gadd45a-IRES-EGFP | Forward (+pCLEG) | GTGGTGGTACC*AGATCT*ATGACTTTGGAGGAATTCTCGGCTGCA |
|  |  | Reverse (+pCLEG) | GAAGCTTGAG*CTCGAG*TCACCGTTCCGGGAGATTAATCA |
| 2 | Gadd45a-AU1 (CT)-IRES-EGFP | Forward (+pCLEG) | GTGGTGGTACC*AGATCT*ATGACTTTGGAGGAATTCTCGGCTGCA |
|  |  | Reverse (AU1+pCLEG) | GAAGCTTGAG*CTCGAG*TCA**AATATAGCGATAGGTATC**GCCGCCCCGTTCCGGGAGATTAATCA |
| 3 | Gadd45a-AU1 (NT)-IRES-EGFP | Forward (pCLEG+AU1) | GTGGTGGTACC*AGATCT*ATG**GATACCTATCGCTATATT**GGCGGCATGACTTTGGAGG  AATTCTCGGCTGCA |
|  |  | Reverse (+pCLEG) | GAAGCTTGAG*CTCGAG*TCACCGTTCCGGGAGATTAATCA |
| 4 | R-Gadd45a-AU1 (NT)-IRES-EGFP | Forward (a24g_t27c_a30t_a33t_a36g) | tgtgtgctggtgacgaac**ccgcactcttctcagtggaag**gatcctgccttaag |
|  |  | Reverse (a24g_t27c_a30t_a33t_a36g) | cttaaggcaggatcc**ttccactgagaagagtgcggg**ttcgtcaccagcacaca |
| 5 | Mouse Gadd45a | Forward | TGCGAGAACGACATCAACAT |
|  |  | Reverse | TCCCGGCAAAAACAAATAAG |
| 6 | Mouse beta-actin | Forward | GATCATTGCTCCTCCTGAG |
|  |  | Reverse | CTCATCGTACTCCTGCTTGCT |

**Table S1. Primers used to generate different Gadd45a constructs and for RT-PCR analysis.**

For constructs 2-4, oligonucleotide PCR primers included an AU1 epitope tag (DTYRYI, codons bolded) while primers for constructs 1-3 included BglII/XhoI restriction sites (italics). Amplified products were cloned into pCLEG vector. Primers for 5 and 6 were used for RT-PCR analysis.
